# Stereotactic body radiation therapy for reirradiation of localized adenocarcinoma of the pancreas

**DOI:** 10.1186/1748-717X-7-74

**Published:** 2012-05-18

**Authors:** Chris E Lominska, Keith Unger, Nadim M Nasr, Nadim Haddad, Greg Gagnon

**Affiliations:** 1 Department of Radiation Oncology, University of Kansas Medical Center, MS 4033, 3901 Rainbow Blvd, Kansas City, KS, 66160, USA; 2 Department of Radiation Medicine, Georgetown University Hospital, Georgetown, USA; 3 Virginia Hospital Center Radiation Oncology, Arlington, VA, USA; 4 Division of Gastroenterology, Department of Internal Medicine, Georgetown University Hospital, Georgetown, USA

**Keywords:** SBRT, Reirradiation, Radiotherapy, Pancreatic cancer

## Abstract

**Background:**

Local control rates are poor in the treatment of pancreatic cancer. We investigated the role of hypofractionated stereotactic body radiation therapy (SBRT) for salvage or boost treatment after conventional doses of external beam radiation therapy.

**Methods:**

All patients treated with SBRT for pancreatic adenocarcinoma at Georgetown University from June 2002 through July 2007 were examined. Eligible patients had prior external beam radiation therapy to the pancreas. Treatment parameters and clinical and radiographic follow-up were evaluated.

**Results:**

Twenty-eight patients were identified who received SBRT after a median prior external beam radiotherapy dose of 50.4 Gy. The median patient age was 63 years old and the median follow-up was 5.9 months. Twelve of fourteen (85.7%) evaluable patients were free from local progression, with three partial responses and nine patients with stable disease. Toxicity consisted of one case of acute Grade II nausea/vomiting, and two cases of Grade III late GI toxicity. The median overall survival was 5.9 months, with 18% survival and 70% freedom from local progression at one year.

**Conclusions:**

Hypofractionated SBRT reirradiation of localized pancreatic cancer is a well-tolerated treatment. Most patients are free from local progression, albeit with limited follow-up, but overall survival remains poor.

## Introduction

Poor local control and frequent distant failure are problematic aspects of the management of pancreatic cancer. Although conventional radiation techniques may be employed for local control, they incur toxicity and interrupt use of full dose gemcitabine, the most active systemic agent for the disease. Radiation doses are limited by the presence of critical normal structures, which include spinal cord, small bowel, stomach and kidneys. The emerging technology of stereotactic body radiation therapy (SBRT), as an adjunct or alternative to conventional radiation techniques, offers the potential for radiation dose escalation, retreatment, and/or decreased interruption of systemic therapy. The safety, efficacy and technical aspects of this treatment modality have not been fully defined.

In the absence of surgical resection, cancer of the pancreas is considered a uniformly lethal disease. After surgery local recurrence rates are estimated to range from 50-75% [1,2]. Chemotherapy is recommended for patients able to tolerate treatment, but response rates for gross disease are poor. The role of radiotherapy is controversial in both the adjuvant (post-operative) and definitive (unresectable localized disease) settings. Supporters of adjuvant radiotherapy point to the landmark Gastrointestinal Tumor Study Group (GITSG) study, where a survival benefit was demonstrated with the addition of adjuvant chemoradiation and maintenance chemotherapy versus surgery alone [3]. Detractors of adjuvant radiotherapy counter with the apparent detrimental effect of chemoradiation from the European Study Group for Pancreatic Cancer (ESPAC-1) [4]. The underpowered European Organization for Research and Treatment of Cancer (EORTC) trial has been interpreted as both undermining the role of adjuvant radiation therapy [5] and supporting it [6].

Recently interest has developed in the use of stereotactic body radiation therapy (SBRT), which involves the delivery of 1–5 fractions with a high dose per fraction delivered with image guidance [7]. The conformity and rapid dose fall-off associated with SBRT offer the potential for dose escalation [8]. SBRT also offers the potential for retreatment of previously irradiated pancreas cancer. This study examines the use of SBRT for reirradiation of locally recurrent/progressive pancreatic cancer.

## Materials and methods

### Patients & eligibility

This is a retrospective study of all patients who received prior external beam radiation therapy and were treated with SBRT at Georgetown University Hospital from June 2002 to July 2007. Approval to review records was obtained from the Georgetown University Hospital institutional review board. Eligible patients had histological demonstration of adenocarcinoma of the pancreas with unresectable locally recurrent or progressive disease, as demonstrated by biopsy or imaging, and no distant metastases. Disease recurrence or progression had to be limited to the primary tumor or regional nodes within the prior radiation field. Patients with invasion of the duodenum or stomach were not eligible for treatment. Twenty-eight eligible patients were identified, 11 of whom were treated with a hypofractionated boost with SBRT delivered within 2 months of completing external beam radiation therapy. The remaining 17 patients underwent salvage SBRT after imaging demonstrated local recurrence/progression. Patient characteristics are summarized in Table 1.

**Table 1:** Summary of patient characteristics

**Characteristic**	**Number of patients (%)**
Tumor location	
Head	16 (57)
Neck	1 (4)
Body	6 (21)
Tail	1 (4)
Lymph node	4 (14)
Age, years	
Median	63
Range	37-87
Sex	
Male	20 (71)
Female	8 (29)
Treatment intent	
Boost (Initial diagnosis)	11 (36)
Salvage (Local recurrence/progression)	17 (61)
Prior radiotherapy dose (Gy)	
Median	50.4
Range	41.40-70.20
Prior local treatment	
Chemoradiation only	20 (71)
Surgery + chemoradiation	8 ( 29)

### Treatment planning & delivery

SBRT was delivered via the CyberKnife, an image-guided frameless stereotactic robotic radiosurgery system (Accuray Corporation, Sunnyvale CA) consisting of a linear accelerator mounted on a robot arm with 6 degrees of freedom [9]. In this system, the confluence of a large number of non-isocentric pencil beams permits the treatment of irregularly shaped target volumes with rapid dose falloff. Twenty-three of the 28 patients had fiducials implanted using endoscopic ultrasound (21 patients), as described previously [10], or CT guidance (2 patients). For these patients respiratory motion was accounted for using fiducial tracking via the Synchrony^TM^ system (Accuray Corporation, Sunnyvale CA). For the remaining five patients, the tumor recurrence was felt to have limited respiration-related motion (e.g., retroperitoneal recurrence). In these cases, spinal tracking was used without compensation for respiratory motion.

Treatment planning began with acquisition of a CT with IV and PO contrast (23/28 patients) approximately 7 days after fiducial implantation. Scan were acquired with free breathing with no abdominal compression or 4D CT techniques used. Gross tumor volume (GTV) was delineated as the radiographically evident gross disease by contrast CT or PET/CT. In select cases (5/28 patients), the GTVs were manually contoured on PET/CT, which was obtained in the treatment position, and fused with the treatment planning CT images. At the discretion of the treating physician, a clinical target volume encompassing areas of potential subclinical disease spread was also designated. In most cases the CTV was the GTV. The expansion from CTV to planning target volume (PTV) was 0–5 mm. As appropriate, organs at risk were contoured including stomach, duodenum, kidney, and spinal cord. Primary organs at risk were the duodenum and/or stomach. These were restricted to a max point dose less than or equal to the prescription dose. Kidney and spinal cord were also evaluated but the gastrointestinal structures were the primary planning objective. Treatment dosimetry parameters were calculated using the formula: conformity index = prescription isodose volume / target volume and homogeneity index = maximum dose / prescription dose [11]. SBRT treatment characteristics are listed in Table 2. A variety of fractionated treatment schemes were employed as follows: 30 Gy in 5 fractions (1 patient); 27.5 Gy in 5 fractions (2 patients); 25 Gy in 5 fractions (1 patient); 24 Gy in 3 fractions (10 patients); 22.5 Gy in 3 fractions (3 patients); 21 Gy in 3 fractions (9 patients); 20 Gy in 5 fractions (2 patients).

**Table 2:** Treatment characteristics

**Treatment Parameter**	**Median (range)**
Total SBRT dose (Gy)	22.5 (20–30)
SBRT dose per fraction (Gy)	7 (4–8)
Number of fractions	3 (3–5)
Isodose line	75% (60-90%)
Max point dose to stomach (Gy)	20 (10–30)
Max point dose to small bowel (Gy)	20 (13–30)
Homogeneity index	1.3
Conformity index	1.6
Target volume (mL)	44 (16–198)
Volume receiving prescription dose (mL)	69 (22–223)

All patients had previously undergone chemotherapy either with 5-fluorouracil (5-FU) based therapy, gemcitabine, or both. Prior chemotherapy agents also included targeted agents, platinum compounds and taxanes. Two of the patients had concurrent capecitabine with SBRT; no patients had gemcitabine within two weeks before or after SBRT. Patients were treated on consecutive weekdays in most cases, and treatment was completed within two weeks.

### Follow-up & statistical analysis

Follow-up information was obtained from chart review of imaging studies, survival information from clinical follow-up and the social security death index. Toxicity was graded according to the Common Terminology Criteria for Adverse events, version 3.0. Survival was calculated from the date of beginning SBRT. Local control was evaluated by CT or PET/CT. Patients were classified as free from local progression if the radiologist reported stable or decreased tumor size or FDG-activity; local failures were denoted if the radiologist reported tumor progression by increase in tumor size or FDG-activity. For CT scans, RECIST criteria were used. For PET scans, response was classified subjectively based on the impresson of the radiologist. Due to the extent of prior treatment and the difficulty of distinguishing inflammatory changes from residual tumor, no patients were classified as complete responses. Kaplan-Meier analysis was used for estimates of local control and overall survival.

## Results

**Figure 1:**
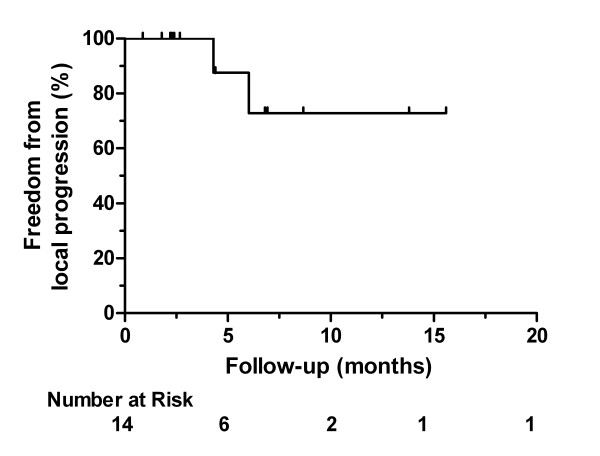
Actuarial plot of freedom from local progression from date of stereotactic body radiotherapy.

Clinical follow-up was available for all patients and radiological follow-up was available for 14 patients (Figure 1). The median duration for any follow-up was 5.9 months (range, 1–27 months), and the median radiographic follow-up was 3.5 months (range, 1–16 months). Freedom from local progression was obtained in 12/14 patients (86%). At last radiological follow-up, 6 (43%) patients were free from local progression and distant progression, 6 (43%) patients were free from local progression but experienced distant failure, and 2 (14%) patients had both local and distant progression by CT scan. Of the 6 patients with local control, 3 (50%) were partial responders (two by PET and one by CT) and the remainder (50%) were locally stable. For the two patients with PET/CT scans, FDG avidity resolved but there was a residual mass on CT, and they were considered to have a partial response. The response rate for all patients with radiographic follow-up was 21%.

**Figure 2:**
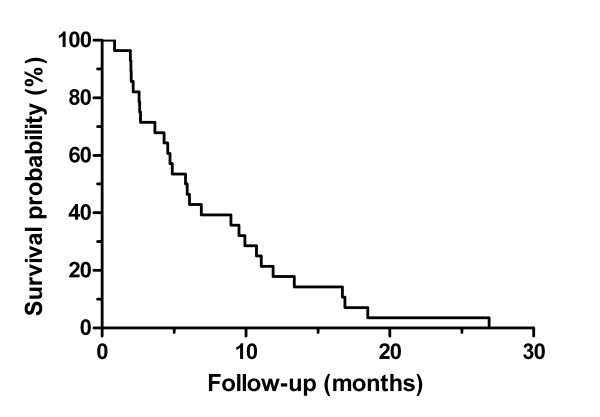
Actuarial plot of survival from date of stereotactic body radiotherapy.

At the time of last follow-up in June 2008, all patients had died. Overall survival is shown in Figure 2. Median overall survival was 5.9 months from the date of SBRT treatment, ranging from 1–27 months. Eleven patients (39%) had 9 months or greater survival. Survival at one year was 18%.

Treatment was well tolerated; acute toxicity consisted of Grade I fatigue and nausea, with the exception of one patient who required IV hydration for Grade II nausea and vomiting while on treatment, but completed treatment without delay. There were two late Grade III GI complications. In one case a patient suffered a bowel obstruction which resolved with conservative management. The second patient had a gastric perforation which resolved with conservative management. Both patients were treated with 21 Gy in three fractions, and had received 50.4 Gy to the abdomen previously. For the patient with a gastric perforation, the maximum point dose to the stomach was 10 Gy; the maximum point dose to the small bowel was 16 Gy in the patient with a bowel obstruction. Given the mobility of these organs at risk between or even during treatments, uncertainty about the actual dose delivered to these organs is inevitable. Qualitiative review of the plans did not reveal a difference in dose to or volume of the critical structures in patients with or without complications. It is now the practice of the authors to use five fractions, which potentially lowers the dose received by a mobile organ at risk.

## Discussion

The current study examines the use of fractionated SBRT for treatment of pancreatic adenocarcinoma in the setting of prior conventionally fractionated external radiotherapy. Our interest in the use of SBRT for reirradiation of pancreas cancer arises from the difficulty of obtaining local control in this disease. Even in the setting of modern multi-modality therapy (surgery and gemcitabine-based chemotherapy or chemoradiation) local recurrence remains frequent [12,13]. The RTOG 97–04 trial demonstrated local recurrence rates of 23% (gemcitabine arm) to 28% (5-FU arm) with the administration of 50.4 Gy adjuvant chemoradiation [13]. In patients with unresectable local disease, response rates to either chemoradiation or chemotherapy are poor. For example, in a recent publication from the Eastern Cooperative Oncology Group (ECOG) reporting gemcitabine plus radiotherapy versus gemcitabine alone, the response rate was 5% for chemotherapy alone and 6% for chemoradiation, although rates of stable disease were higher with the addition of radiation (35% vs 68%) [14]. The potential benefits of SBRT include decreased treatment time, dose intensification to gross disease, and minimal interruption of systemic therapy [15]. These must be weighed against the disadvantages, which include the potential for increased toxicity, principally gastrointestinal, the prevalence of distant failure negating any theoretical survival advantage for local control, and the difficulty in demonstrating long-term local control or palliative benefit given the overall poor prognosis of these patients.

Investigation of SBRT for pancreas cancer has included a series of studies from Stanford [8,15-17]. A Phase I dose escalation study demonstrated the tolerability of 25 Gy delivered in a single fraction, producing 100% local control in all 6 patients treated, with a median follow-up of 4.5 months [16]. The prevalence of distant failure negated any survival benefit and demonstrated the ongoing importance of systemic therapy in this disease. A phase II trial investigated the use of 5-FU based chemoradiation with 45 Gy delivered via intensity modulated radiation therapy to the tumor and regional lymph nodes, followed by a 25 Gy SBRT boost [8]. Nineteen patients were enrolled, of whom sixteen underwent SBRT. Two patients experienced Grade III GI toxicity (gastroparesis), and additional patients developed duodenal ulcers. Although local control was favorable at 94%, distant progression remained problematic, and in conjunction with the increase seen in side effects, prompted discarding of the protocol. Systemic therapy was intensified in the subsequent Phase II trial which incorporated a single fraction 25 Gy SBRT treatment into a regimen of gemcitabine chemotherapy [17]. Sixteen patients were treated with full-dose gemcitabine, with SBRT performed between cycles one and two, followed by more chemotherapy until progression or tolerance was reached. Only three patients failed locally, but distant progression remained frequent (15/16 patients) and overall survival was 11 months. Late gastrointestinal toxicity remained problematic with five patients experiencing Grade II, and two patients Grade III/IV toxicity. The Stanford studies [8,16,17] demonstrate potentially encouraging rates of local control when taken together, but the prevalence of distant progression prompted additional chemotherapy and external beam radiotherapy, with a concerning concomitant increase in toxicity. The durability of local control is difficult to characterize given poor overall survival. The Stanford experience has been recently summarized in a publication of 77 patients, with freedom from local progression rates of 84% at 1-year, and a 1-year overall survival rate of 21% [15].

Other reports of SBRT for pancreatic cancer include a phase II trial of 22 patients from Denmark that showed concerning levels of acute (79% Grade II or greater) and late (22% severe, not further defined by authors) GI toxicity with a poor local control rate of 57% [18]. A caveat to these results is that an abdominal compression system was used for treatment delivery, and the prescription was 45 Gy in three fractions to the clinical target volume, which included the tumor and surrounding edema. An expansion of 0.5 to 1 cm created a planning target volume with an average size of 136 mL which was treated to a minimum dose of 30 Gy in three fractions. A series of 36 patients who underwent SBRT for locally advanced pancreatic cancer has recently been reported from Beth Israel [19]. Patients with unresectable, non-metastatic disease underwent 3 fractions of SBRT followed by gemcitabine chemotherapy. Dose to the small bowel was limited to less than 10 Gy per fraction; the prescribed tumor dose varied (24 Gy in 3 fractions, 30 Gy in 3 fractions, or 36 Gy in 3 fractions) depending on the separation between the tumor and duodenum. The local control rate was 78% with a median overall survival time of 14.3 months. Toxicities included 9 acute Grade II, 3 acute Grade III events, and 2 late Grade III events attributed to SBRT. The University of Pittsburgh has reported a series of 71 patients who underwent SBRT for locally advanced adenocarcinoma of the pancreas for indications including locally unresectable disease (40 patients), local recurrence (11 patients), metastatic disease (8 patients) and positive margins after resection (12 patients) [20]. Sixty-seven patients were treated with a single fraction of 18–25 Gy and the remainder received fractionated treatment. Freedom from local progression was 65% overall and 49% at one year with a median overall survival of 10 months. Grade I-II GI toxicity was found in 40% of patients but higher grade toxicity was minimal, consisting of 3 patients with acute Grade III GI toxicity. The planning volume was gross disease with a 2 mm margin, and the median tumor volume was 17 mL.

Descriptions of SBRT in the treatment of previously irradiated pancreas cancer are scarce in the literature. There are 16 cases described in the Stanford boost trial [8]. Fifteen patients in the Pittsburgh series had received prior radiotherapy to a median dose of 45 Gy [20]. However, the Pittsburgh study did not report outcomes separately for the patients with prior radiation, limiting the conclusions that can be drawn about the use of SBRT in the setting of prior radiation. Seo et al retrospectively reported the largest series after external beam radiotherapy (40 Gy in 20 fractions) as a planned boost [21]. 15–17 Gy was delivered as a single fraction. One year local progression free survival was 70% with 25 patients evaluable. One Grade IV late bowel obstruction was seen.

Reirradiation is a clinically relevant subject of investigation given that approximately one-fourth of all patients who undergo optimal trimodality therapy suffer from local failure. For patients with residual or recurrent localized disease after definitive treatment, current treatment options are limited. Response rates are poor with chemotherapy alone and surgical resection is possible in only highly selected cases. Though patients also have significant risk for development of regional or distant disease, local progression may cause significant morbidity. SBRT has been shown to provide excellent local control rates when used in the definitive setting. The minimal toxicity and short treatment duration of SBRT allow for the early resumption of systemic chemotherapy. Our finding of 70% freedom from local progression at one year is similar to the Stanford series (84% at one year) [15] and more encouraging then the Danish results [18]. However, interpretation of local control is limited by the patients lost to radiographic follow-up, the difficulty of interpreting CT findings, and short follow-up secondary to distant progression and patient mortality. Additional information may be gained by increasing use of PET/CT [22]. Anecdotally, the current series includes two patients who had prolonged PET responses (nine months to a year) after salvage SBRT despite residual CT masses, who remained free of local disease progression without additional therapy. Both ultimately failed in the peritoneum.

Interpretation of our results includes consideration of the limitations inherent to a retrospective review. These include selection bias, patient heterogeneity, the use of SBRT as either boost or salvage, the use of a variety of fractionation schemes, and exposure to a range of chemotherapies. Additionally, our radiographic follow-up is limited, making interpretation of freedom from local progression difficult. Distant failure remains problematic and survival poor. More detailed evaluation of palliative response, including quality of life measures, would be informative in future studies. Given the poor prognosis for these patients and the limited responses to the available treatment options, we feel our series provides valuable information about an acceptable level of toxicity for additional local treatment in these heavily previously treated patients.

A disparity exists in the reported toxicities for SBRT. The Pittsburgh [20], Beth Israel [19], and Stanford single SBRT alone and boost trials [8,16] suggest Grade III and higher toxicities are less than 10-15% with SBRT. The Danish study [18] and Stanford SBRT with gemcitabine study [17] suggest significantly higher rates of toxicity. The current series suggests that with modest hypofractionation of SBRT (3–5 fractions) to limited volumes, treatment can be delivered with acceptable acute and late GI toxicity (less than 10%) in the setting of prior chemotherapy and definitive doses of external beam radiotherapy. The short follow-up limits interpretation of late toxicity. We report detailed evaluation of treatment plans, including treated volumes, conformity and homogeneity indices, and doses to critical structures that should prove useful to other investigators wishing to evaluate SBRT programs for pancreas cancer. The authors favor fractionation with SBRT in pancreas cancer given that increasing dose per fraction is associated with increased late effects. Because the small bowel is a mobile structure mounted on the mesentery it has the potential to move in or out of the treatment field between fractions; in effect, feathering the dose to any given portion of bowel.

## Conclusion

Our findings demonstrate acceptable toxicity and potentially encouraging freedom from local progression with fractionated SBRT for pancreas cancer in the setting of prior chemotherapy and radiotherapy. Given the high rates of distant progression, improvements in systemic therapy remain desirable. SBRT can be interdigitated with minimal interruption in full dose systemic therapy. Future investigation might include the use of novel systemic agents or radiosensitizers, rigorous quality of life assessment of palliative benefit, and incorporation of metabolic tumor information. It is our practice to observe selected patients with local recurrence for a period of time while on systemic therapy, and in the absence of evidence of systemic failure, to offer SBRT after 3–6 months of localized disease with the goal of improving local control, while minimizing local overtreatment of patients with occult metastatic disease.

## Abbreviations

SBRT, Stereotactic body radiation therapy; GTV, Gross tumor volume; CTV, Clinical target volume; PTV, Planning target volume.

## **Competing interests**

Dr. Gagnon has received grant funding, speakers honoraria, and has served on the clinical advisory board for Accuray, Inc., Sunnyvale, California.

## **Authors’ contributions**

CEL, KU, NMN, NH,GG, participated in the design of study and analysis of data and reviewed and approved the final manuscript.
